# The Impact of Angiotensin Converting Enzyme-2 (ACE-2) on Bone Remodeling Marker Osteoprotegerin (OPG) in Post-COVID-19 Iraqi Patients

**DOI:** 10.7759/cureus.29926

**Published:** 2022-10-04

**Authors:** Islam S AL-Azzawi, Nawar S Mohammed

**Affiliations:** 1 Clinical Biochemistry, University of Baghdad, Baghdad, IRQ; 2 Biochemistry, University of Baghdad, Baghdad, IRQ

**Keywords:** dexa scan, osteoporosis, opg, ace-2, covid-19

## Abstract

Introduction

As COVID-19 affects human genes in several types of peripheral tissue, numerous disorders occur after recovery. The virus enters host cells via angiotensin-converting enzyme-2 (ACE-2) receptors that affect bone remodeling, leading to osteopenia or osteoporosis, which is characterized by low bone mineral density (BMD). The adult skeleton undergoes about 10% remodeling annually, which is crucial for preventing fatigue damage and preserving calcium homeostasis. An imbalance in the rates of bone production and resorption causes bone loss. Osteoprotegerin (OPG) is one of the regulators involved in the bone remodeling mechanism, it decreases the activity of NF-B receptors that activates the receptor activators of NF-B ligand (RANKL) pathway, which maintains the bone homeostasis balance. This study aims to find out the disruption of bone homeostasis balance in Iraqi post-COVID-19 patients.

Materials and methods

It is a case-control study that includes 130 Iraqi subjects enrolled. They were divided into two groups - the first group consisted of 80 post-COVID-19 infection patients, while the second group consisted of 50 who were not infected with COVID-19. Also, the levels of ACE-2 and OPG were measured in the serum by the ELISA technique. The BMD was measured by the DEXA scan technique.

Results

This study found that there is an effect of coronavirus infection on the bone strength measured by the Mean of the OPG level, which was found to be highly significant in the serum of post-COVID-19 patients when compared with non-COVID-19 subjects (P-value = 0.001), but the Mean of ACE-2 level was statistically non-significant between the two groups (P-value = 0.13). Also, the BMD of post-COVID-19 patients that was measured by DEXA scan had a statistically highly significant T-score% between the two groups.

Conclusion

The current study found that there was an effect of COVID-19 on the bone remodeling mechanism, which may be causing osteopenia or osteoporosis for Iraqi subjects enrolled in the current study. Also, analyzing the OPG level in the serum could be helpful in predicting low BMD.

## Introduction

COVID-19, which is characterized by a high cytokine influx, is an acute respiratory tract infection, which the WHO declared a pandemic in March 2020 [1]. This virus impacts the human body after recovery. The virus penetrates the host cells via angiotensin-converting enzyme-2 (ACE-2) receptors, which are found on different types of peripheral tissue in the human body, such as the kidneys, liver, lungs, and skeletal system, which may lead to disorders in multiple organs [2,3].

The adult skeleton undergoes about 10% remodeling annually, which is crucial for preventing fatigue damage and preserving calcium homeostasis. An imbalance in the rates of bone production and resorption causes bone loss [4]. The bone is an extremely dynamic organ that is continuously remodeling, which is a mechanism for maintaining the serum calcium and phosphate levels by replacing old bone cells with new bone cells [5]. There are four types of bone cells: osteoblast cells, active osteoblasts, osteoclast cells, and osteocytes [6]. Osteoblast cells, play a role in the production of bone matrix by the production of many proteins for the organization of bone metabolism [7]. Bone lining cells, regulate cancellous bone thickness and surface area [8]. Osteoclast cells are capable of resorbing bone by dissolving minerals, digesting the bone matrix, and producing hydrochloric acid [9]. Meanwhile, multiple cytokines have been implicated in osteoclast formation [10]. And osteocytes, which represent 95% of the total cell count in bone tissue, play a role in activating the osteoclasts and osteoblasts for bone remodeling mechanisms [11].

The remodeling cycle occurs in cortical and trabecular bone in a highly regulated and stereotyped manner, with five overlapping processes occurring over a 120-200-day period, with resorption lasting around two weeks [12]. The reversal phase, which lasts four to five weeks and sees bone resorption switch to creation, can take up to four months. Coronavirus infection may have an effect on bone remodeling [13]. Osteoprotegerin (OPG) is a soluble glycoprotein consisting of 380 amino acid peptides that are produced by B lymphocytes and also produced by osteoblasts themselves, which suppresses osteoclast formation [14].

OPG is considered one of the regulators of bone remodeling mechanisms by neutralizing the activity of the receptor activators of NF-B ligand (RANKL) [15]. Higher circulating OPG levels in the blood are frequently reported in osteoporotic patients and are typically interpreted as a consequence of accelerated bone turnover and a compensatory reaction to excessive osteoclast activity [16].

Osteoporosis is a condition characterized by low bone mineral density (BMD) which causes a decline in bone strength [17]. Osteoporosis may be caused by an immune system-bone turnover interaction, which increases the risk of fragility fractures [18]. The current study's goal was to determine the bone homeostasis balance in Iraqi post-COVID-19 patients and to identify the effect of the viral infection on both osteoclastogenesis and osteoblastogenesis by detecting the change in OPG levels in their blood.

## Materials and methods

This research is a case-control study, established from November 20, 2021 to March 2, 2022, and the Research Protocol was approved by the Iraqi Ministry of Health & Environment (approval number 02/2021). There were 130 subjects who were enrolled in this study. They were divided into two groups. The first group included 80 patients (diagnosed to have COVID-19 by PCR testing or a chest CT scan) three months ago and who have made a full recovery. The second group included 50 subjects that have not been infected with the coronavirus. All the subjects were taken from Al Yarmouk Teaching Hospital and the Shifa Center City of Medicine Hospital. Their age range was between 18 to 45 years old for women and 18 to 60 years old for men. The serum ACE-2 was measured using a commercial enzyme-linked immunosorbent assay ELISA technique provided by the Human ACE-2 ELISA Kit made in China. The serum OPG levels were also measured by using the ELISA technique provided by the Human OPG ELISA Kit by MyBioSource Inc. (made in China). The BMD was measured by dual-energy X-ray absorptiometry (DEXA scan). The World Health Organization (WHO) has established standards for the diagnosis of osteoporosis based on the accuracy and repeatability of DEXA scans [19]. T-scores are used in the diagnosis of osteoporosis, a T-score of less than 1.0 is considered osteopenia, and lower than -2.5 is considered osteoporosis [20].

All subjects with hyperparathyroidism or hypoparathyroidism, early menopausal or postmenopausal women, subjects with chronic renal disease, and subjects who were pre-diagnosed as osteopenic or osteoporotic patients were excluded from this study.

Statistical analysis

The data were analyzed using the Statistical Package for Social Sciences (SPSS) version 26 (IBM Corp., Armonk, NY). And the data were presented as mean with standard deviation. The correlation between continuous variables was evaluated using Pearson's correlation test (r), in accordance. Statistical significance was defined as a P-value of 0.05 or less [21].

## Results

There were significant differences in the mean ± SD of OPG levels in serum between the two groups with a P-value of 0.001 (P-value of 0.05 is considered statistically significant).

There were no significant differences in the mean ± SD of ACE-2 levels between the two groups (P-value = 0.13). The group of post-COVID-19 patients had elevated serum levels of the bone turnover marker OPG versus non-COVID-19 subjects with a statistically significant P-value (= 0.001). The bone mineral density was found to have a statistically significant DEXA (T-score %) (P-value = 0.001), as shown in Table 1.

**Table 1:** The comparison of OPG level, ACE-2 level, and DEXA score (%) for the two groups **Statistically highly significant; OPG, Osteoprotegerin; ACE-2, Angiotensin-Converting Enzyme-2; DEXA, Dual-Energy X-ray Absorptiometry

Biochemical markers	COVID-19 patients Mean ± SD (n =80)		Non-COVID-19 subjects Mean ± SD (n =50)	P-value
ACE-2 (pg/mL)	173.25 ± 36.7	185.74 ± 50.0		0.13
OPG (ng/mL)	2.24 ± 1.0	0.7 ± 0.21		0.001**
DEXA (T- score %)	- 0.43 ± 0.94	0.45 ± 0.64		0.001**

**Figure 1:**
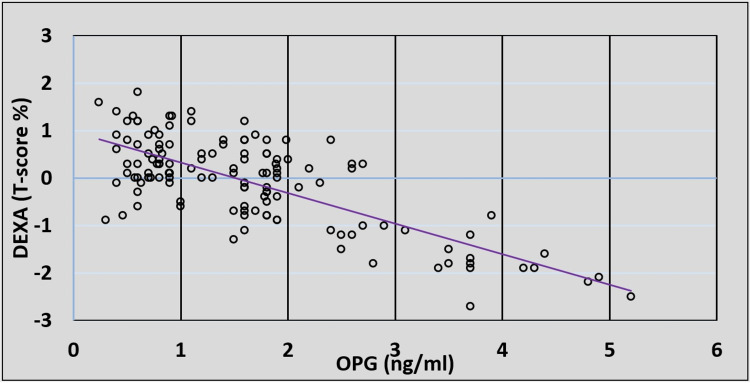
The correlation between serum osteoprotegerin (OPG) (ng/mL) and dual-energy x-ray absorptiometry (DEXA) T-score

There was no correlation between the serum OPG level or DEXA (T-score %) and the serum level of ACE-2, unlike the correlation between the OPG level in the serum and DEXA (T-score %), which was statistically highly significant with a strong negative linear correlation (P-value = 0.001), as shown in Table 2. There was a significant inverse relationship between OPG levels in the serum of post-COVID-19 patients and their DEXA (T-score %) measurement (Figure 1).

**Table 2:** Correlation between biochemical markers **Statistically highly significant; OPG, Osteoprotegerin; ACE-2, Angiotensin-Converting Enzyme-2; DEXA, Dual-Energy X-ray Absorptiometry

Biochemical markers		ACE-2 (pg/mL)	OPG (ng/mL)	DEXA score (%)
ACE2 (pg/mL)	r	-	0.108	- 0.98
	P-Value	-	0.22	0.266
OPG (ng/mL)	r	0.108	-	- 0.745
	P-Value	0.22	-	0.001**
DEXA (T-score %)	r	- 0.98	- 0.745	-
	P-Value	0.266	0.001**	-

## Discussion

Bone remodeling is a mechanism of bone turnover that destroys the old bone cells by osteoclasts and reforms new bone cells by osteoblasts [22], whereas the role of ACE-2 is related to the renin-angiotensin-aldosterone system (RAAS), which works as an entry receptor for coronavirus [23]. After coronavirus binds to various types of cells, including hematopoietic stem cells (HSC), it can damage several organs in the host body cells by activating the immune system [14]. The result of binding the ACE-2 receptors is a serious complication, leading to increased production of the pro-inflammatory phase, which is called the “cytokine storm” [24]. The OPG that is derived from Bi cells does not contribute to the suppression of bone resorption under normal physiological conditions, but the OPG that is produced by mature osteoblasts can lead to osteoporosis [25]. For that reason, OPG, which has the main role in the bone turnover mechanism, can increase as a homeostatic mechanism to minimize bone damage and work as an osteoclastogenesis inhibitor to prevent osteoporosis [26]. The level of OPG increases in the blood due to reduced bone mass and microstructural degeneration of bone tissue, which are the markers of osteoporosis, a degenerative skeletal condition that increases bone fragility and fracture susceptibility [4].

Other researchers discovered that the virus has an effect on the skeletal system during coronavirus infection and that ACE-2 plays a role in osteoblast and osteoclast synthesis by reducing bone resorption in the Mas receptor (MasR) pathway, which is a class of G-protein-coupled receptor [27]. Huang et al. discovered that inflammatory cytokines such as IL-1, IL-6, TNF-, G-CSF, IP-10, MCP-1, and MIP-1 are increased during coronavirus infection and affect the osteoclastogenic pathway by inhibiting osteoblast and OPG levels and increasing osteoclast hypoxia, affecting bone activity [28]. The corticosteroid treatment that is administered during the infection can also lead to bone damage, which is considered one of the factors leading to the prevalence of osteopenia or osteoporosis [29].

On the other hand, elevated levels of C-reactive protein (CRP), which is due to the cytokine storm with low levels of cholecalciferol (vitamin D) in the body and low levels of estrogen hormone together with steroid therapy, can have an effect on the bone remodeling mechanism, which leads to osteopenia or osteoporosis [1].

Limitations

There are certain limitations in the current study, including the nature of the study and the relatively small sample size. This is due to the difficulty to confirm previous coronavirus infection as no medical history is available for each subject. For this reason, the current study excluded any subjects who did not have CT scans or PCR results to confirm the coronavirus infection. As a result, the number of enrolled subjects was limited.

This study was a case-control study, the two groups were matched in age and BMI range. The control group was selected according to their medical history with no previous coronavirus infection (according to their PCR and/or CT scan) and no recorded history of low BMD. Any subjects with previous steroid therapy were excluded from the current study for the two groups.

## Conclusions

COVID-19 enters host cells via ACE-2 receptors because it works on human genes in various types of peripheral tissue in the human body, this may have effects on bone remodeling, which is characterized by low BMD. An imbalance in the rates of bone production and resorption causes bone loss. OPG is a bone remodeling pathway regulator that reduces the activity of NF-B receptor activators (RANKL) while preserving bone homeostasis in the human skeletal system. The current study found that there was an effect of COVID-19 on the bone remodeling mechanism, which may be causing osteopenia or osteoporosis for Iraqi subjects. Also, analyzing the OPG level in the serum could be helpful in predicting low BMD.
